# Starting at the endophenotype: A role for alpha-CaMKII in schizophrenia?

**DOI:** 10.1186/1756-6606-1-5

**Published:** 2008-09-10

**Authors:** Paul W Frankland, Masanori Sakaguchi, Maithé Arruda-Carvalho

**Affiliations:** 1 Program in Neurosciences and Mental Health, The Hospital for Sick Children, Toronto, M5G 1X8, Canada; 2 Department of Physiology, University of Toronto, Toronto, M5S 1A8, Canada; 3 Institute of Medical Science, University of Toronto, Toronto, M5S 1A8, Canada

## Abstract

Using an endophenotype-driven screen, a new study finds that α-calcium/calmodulin kinase II mutant mice exhibit a range of behavioral abnormalities related to schizophrenia. Perhaps most strikingly, this cluster of schizophrenia-related endophenotypes was associated with abnormal neurogenesis in the adult hippocampus, raising the possibility that disrupted adult neurogenesis lies at the core of this and other psychiatric disorders.

## Editorial

Schizophrenia is a chronic, debilitating form of mental illness, affecting more than 1% of the adult population [1]. Current treatments are palliative at best – reducing the severity of symptoms rather than providing a cure – in part, because of the paucity of our understanding of the molecular bases of this complex, polygenic disorder. A more comprehensive understanding of the molecular underpinnings of schizophrenia is an essential foundation for the development more effective treatments (and eventual cure).

A new study by Miyakawa and colleagues [this issue, Molecular Brain 2008, 1:6] sheds fresh light on potential molecular mechanisms underlying schizophrenia. In their study, the authors ran different lines of mutant mice through a comprehensive behavioral test battery in order to screen for behavioral abnormalities relevant to schizophrenia. In such a phenotypic screen, there are two critical decisions: First, which behaviors to focus on? As is the case with many psychiatric disorders, schizophrenia is characterized by a bafflingly heterogeneous collection of behavioral symptoms. These include both positive symptoms (e.g., hallucinations, delusions, disordered thought) and negative symptoms (e.g., anhedonia, decreased motivation, attentional problems and impaired working memory). Clearly, some of these abnormalities are uniquely human and impossible to model in a mouse (even for the most adept mouse psychiatrist). The solution, then, is to focus on a subset (rather than the entire constellation) of behavioral abnormalities that are nonetheless heritable and readily modeled in mice. Using this endophenotype-driven approach, Miyakawa and colleagues focused on characterizing working memory, anxiety, aggression and infradian and circadian rhythms in their mutant mice.

The second critical decision is which mice to screen? Thousands of knockout, knockin and transgenic lines of mice have been engineered. As screening large numbers of mice is prohibitively time-consuming and expensive, it makes sense to narrow down the field to a more tractable number of players. Here, Miyakawa and colleagues took advantage of their previous work with mice that have a forebrain-specific deletion of calcineurin (CN). They found that these mice exhibited abnormalities in a number of behaviors related to schizophrenia [2], and, therefore, in the present study they decided to screen 7 mouse lines with mutations related to either CN signaling or CN-related mechanisms. Of these 7, the mice with the most striking behavioral phenotype were those carrying a heterozygous null mutation for α-calcium/calmodulin kinase II (α-CaMKII^+/- ^mice). These are mice with a storied history in the field of learning and memory. Originally generated by Alcino Silva while a postdoctoral fellow in Susumu Tonegawa's lab at MIT, initial studies with the α-CaMKII homozygous mutants helped to establish that α-CaMKII plays an essential role in synaptic plasticity and hippocampal learning [3,4]. Subsequent studies using the heterozygous mutants suggested that α-CaMKII also plays a key role in cortical plasticity, and in the consolidation of remote memories [5,6].

In their new study, Miyakawa and colleagues found that the behavioral phenotype of the α-CaMKII^+/- ^mice is more complex (and perhaps more abnormal) than previously appreciated. Perhaps most notably, the α-CaMKII^+/- ^mice have severe working memory deficits (tested either in a radial arm maze or delayed alternation task). Working memory deficits are a core symptom of schizophrenia, and working memory deficits have been consistently identified in other mouse models of schizophrenia (most recently, for example, in mice with mutations in *Disrupted-in-schizophrenia-1 *[*Disc1*] [7,8]). Adding to this collection of psychiatric-related behavioral phenotypes, further analyses revealed that the α-CaMKII^+/- ^mice are unusually aggressive (killing their cage mates given half a chance), less anxious and had dramatically disrupted patterns of daily activity.

Because informative endophenotypes need not be restricted to the behavioral domain, next Miyakawa and colleagues took a look inside the brains of the α-CaMKII^+/- ^mice. Their analyses uncovered quite striking changes in the dentate gyrus of the hippocampus (a brain region that plays a key role in working memory). The dentate gyrus is a special place in the brain – it is one of two regions where new neurons continue to be generated throughout adulthood [9]. Miyakawa and colleagues found that this process – hippocampal adult neurogenesis – was quite abnormal in the α-CaMKII^+/- ^mice. While more new neurons appeared to be produced (proliferation was increased by about 50%), these neurons did not appear to mature normally – the dentate gyrus of α-CaMKII^+/- ^mice contained a higher proportion of granule cells with immature properties (e.g., increased excitability and reduced dendritic branching and length). This shift in balance from mature to immature granule cells suggested to Miyakawa and colleagues that the α-CaMKII^+/- ^mice have an immature dentate gyrus.

That changes in the regulation of neurogenesis in the adult hippocampus are associated with a cluster of behavioral endophenotypes related to schizophrenia in the α-CaMKII^+/- ^mice is particularly noteworthy. There is mounting interest in the potential role of adult neurogenesis in the pathology of a number of psychiatric illnesses, including schizophrenia and depression [10]. For example, antidepressants may well work by increasing hippocampal neurogenesis [11] and, while adult neurogenesis may be unaltered in depressed patients, recent reports suggest that levels of hippocampal neurogenesis are reduced in schizophrenic patients [12]. The emerging hypothesis is that abnormal neurogenesis may contribute to abnormal hippocampal function [10]. Since the hippocampus is involved in regulation of mood and cognition, such aberrant hippocampal processing may cumulatively lead to the sorts of disturbances in thought and mood found in schizophrenia and depression. This is an intriguing new hypothesis and, if true, suggests that in hunting down schizophrenia susceptibility genes it might make most sense to pay special attention to genes that regulate neuronal development and/or adult neurogenesis. In this regard, it is particularly exciting that perhaps the strongest candidate schizophrenia gene – *Disc1 *– is implicated in neuronal migration and differentiation [13]. Consistent with this, α-CaMKII itself regulates neuronal development [14], and the expression of a large number of genes that have been implicated in neuronal development (e.g., genes in the BDNF-MAPK pathway) were differentially regulated in the α-CaMKII^+/- ^mice.

A final thought on the approach that Miyakawa and colleagues adopted in this study. In exploring gene-function relationships both forward-genetic (i.e., phenotype → gene(s)) and reverse-genetic (i.e., gene → phenotype(s)) approaches have traditionally been used [15]. However, there are limitations associated with both approaches. In forward-genetic studies (such as ENU mutagenesis screens) identifying the causative mutation may be an especially time-consuming and expensive process. Similarly, in reverse-genetic studies (such as those using knockout mice) it is necessary to have a good idea about which gene to target and such *a priori *knowledge may often be lacking in complex polygenic disorders like schizophrenia. The strategy used by Miyakawa and colleagues blurs the boundaries between these two traditional classifications. Indeed, the use this hybrid strategy – a phenotypic screen of previously engineered mutant mice – is particularly powerful since it allows for the rapid association of schizophrenia-related endophenotypes with specific genes. A similar strategy has recently been used to identify genes for remote memory [16]. By combining the best of both worlds, Miyakawa and colleagues bring us a step closer to understanding the molecular basis of schizophrenia.
